# Nanocapsules Containing Neem (*Azadirachta Indica*) Oil: Development, Characterization, And Toxicity Evaluation

**DOI:** 10.1038/s41598-017-06092-4

**Published:** 2017-07-19

**Authors:** Tatiane Pasquoto-Stigliani, Estefânia V. R. Campos, Jhones L. Oliveira, Camila M. G. Silva, Natalia Bilesky-José, Mariana Guilger, Johann Troost, Halley C. Oliveira, Renata Stolf-Moreira, Leonardo F. Fraceto, Renata de Lima

**Affiliations:** 1 Programa de Pós-Graduação em Biotecnologia e Monitoramento Ambiental, Universidade de São Carlos (UFSCar), Sorocaba, SP Brazil; 2 Universidade Estadual Paulista (UNESP), Instituto de Ciência e Tecnologia, Laboratório de Nanotecnologia Ambiental, Sorocaba, SP Brazil; 3 Departamento de Bioquímica, Universidade Estadual de Campinas (UNICAMP), Campinas, SP Brazil; 4 LABiToN - Laboratório de Avaliação de Bioatividade e Toxicologia de Nanomateriais, Universidade de Sorocaba (UNISO), Sorocaba, SP Brazil; 5 Departamento de Biologia Animal e Vegetal, Universidade Estadual de Londrina (UEL), Londrina, PR Brazil

## Abstract

In this study, we prepared, characterized, and performed toxicity analyses of poly(ε-caprolactone) nanocapsules loaded with neem oil. Three formulations were prepared by the emulsion/solvent evaporation method. The nanocapsules showed a mean size distribution around 400 nm, with polydispersity below 0.2 and were stable for 120 days. Cytotoxicity and genotoxicity results showed an increase in toxicity of the oleic acid + neem formulations according to the amount of oleic acid used. The minimum inhibitory concentrations demonstrated that all the formulations containing neem oil were active. The nanocapsules containing neem oil did not affect the soil microbiota during 300 days of exposure compared to the control. Phytotoxicity studies indicated that NC_20 (200 mg of neem oil) did not affect the net photosynthesis and stomatal conductance of maize plants, whereas use of NC_10 (100:100 of neem:oleic acid) and NC_15 (150:50 of neem:oleic acid) led to negative effects on these physiological parameters. Hence, the use of oleic acid as a complement in the nanocapsules was not a good strategy, since the nanocapsules that only contained neem oil showed lower toxicity. These results demonstrate that evaluation of the toxicity of nanopesticides is essential for the development of environmentally friendly formulations intended for applications in agriculture.

## Introduction

The global population has increased substantially over the years, generating economic activity and greater demand for food. As a result, there is the need to improve agricultural processes, especially because the expansion of urban areas has decreased cultivable areas worldwide.

There are intense efforts to identify natural products^1–6^, botanical insecticides, and botanical biocides that are able to destroy, render harmless, inactivate, or otherwise control organisms considered damaging to crops^7^.

Botanical biocides, which have been used for over three thousand years^8^, can be incorporated in integrated pest management in commercial crops, including organic production systems^9, 10^. Botanical biocides include essential oils^11^ that were previously employed only as fragrances and are now studied for use as natural pesticides^12, 13^, with great interest in their biological effects^14^. In Asia, the neem tree (“Arishtha”, from Sanskrit, meaning “disease relief”) has been used for centuries in Ayurvedic medicine, one of humanity’s oldest medical systems^15^. In recent times, extracts derived from the tree have been used as natural insecticides, a practice that has increased in the last 30 years, following the isolation of azadirachtin, the main compound present. Azadirachtin presents a broad spectrum of insecticidal action and is biodegradable, not harmful to the environment, and nontoxic to humans, with an oral LD_50_ in mammals of 13,000 mg/kg^16, 17^.

Advantages of the use of botanical biocides include their rapid degradation by sunlight, low persistence in the environment, lower likelihood of the target organism developing resistance, and low residual activity. However, despite these benefits, characteristics such as photosensitivity and rapid degradation are disadvantageous from an agribusiness perspective, since they result in lower efficiency of biocides, relative to conventional pesticides, which necessitates a greater number applications, as well as the fact that biocides rarely present systemic action^18^.

One way to overcome such limitations is to use the types of carrier systems first developed for health applications and widely used in association with drugs, which show promise for reducing the use of pesticides as well as the problems caused by them^19^. The modified release characteristics of these systems have attracted the attention of researchers for the development of formulations that can be loaded with active agents used in agriculture, such as botanical biocides^20^.

Among the different types of carrier systems, polymeric and solid lipid nanoparticles have received attention for agricultural purposes due to their solid matrices, which protect the bioactive compound from degradation and also enable modulation of the release profile^21^. The advantages of polymeric nanoparticles include biocompatibility, biodegradability, the ability to modify and functionalize the surface, incorporation of the active agent without any chemical reactions, and the possibility of modulating the degradation and release of the active agent by selection of the materials used to prepare the nanoparticles^22–25^.

Although carrier systems have been widely studied, little is known about their toxicity towards animals, humans, plants, and the environment. Studies of the toxicity of nanoparticles and isolated carrier systems began in around 2010, and as pointed out by Elsaesser and Howard^26^, there is a need for further research concerning the synthesis of new materials and evaluation of their toxicity. New combinations of materials may exhibit toxic effects different to those observed in earlier studies^27–29^.

Given this background, the present work describes the preparation and characterization of poly(ε-caprolactone) (PCL) nanocapsules loaded with neem oil, for possible use in pest control. Evaluation was made of their cytotoxic and genotoxic potentials, as well as their action on bacteria of the nitrogen cycle and their phytotoxicity towards maize plants (*Zea mays*). The aim was to obtain systems that are less harmful to the environment and human health, and that could contribute to pest control in agriculture.

## Results and Discussion

### Characteristics of the nanoparticles

The results of the physicochemical analyses showed that the nanoparticles had an average size between 410 and 500 nm, polydispersity values below 0.2, and zeta potential varying according to the quantity of neem oil (Table 1S). The nanoparticles were synthesized using polyvinyl alcohol (PVA) as stabilizer, which coated the surfaces of the particles and provided steric stabilization, so surface electrostatic repulsion was not the primary factor influencing colloidal stability. Nanoparticle tracking analysis (NTA) was used to obtain the nanoparticle concentrations, which differed according to the synthesis but showed similar size distributions (Table 1).

**Table 1:** Initial values of the mean diameter (MD, in nm), polydispersity index (PDI), zeta potential (ZP, in mV), and pH for the nanocapsules with or without neem oil (n = 3).

Form.	DLS				NTA	
	MD (nm)	PDI	ZP (mV)	pH	Nanocapsule concentration (mL^−1^)	MD (nm)
NC_20	456.7 ± 4.61	0.129 ± 0.04	+11 ± 1.17	3.67 ± 0.08	4.24 ± 0.236 10^12^	248.5 ± 6.7
NC_15	467.1 ± 5.40	0.144 ± 0.01	−3.47 ± 0.74	3.97 ± 0.08	9.60 ± 0.227 10^12^	244.3 ± 7.3
NC_10	495.8 ± 7.70	0.140 ± 0.05	−16.6 ± 0.36	4.31 ± 0.01	6.30 ± 0.336 10^12^	281.0 ± 6.6
NC	410.7 ± 1.89	0.072 ± 0.03	−27.6 ± 3.90	3.69 ± 0.03	9.05 ± 0.280 10^12^	258.5 ± 2.3

Values expressed as means and standard deviations. NC_20, NC_15, and NC_10 are the formulations with different quantities of neem oil (200, 150, and 100 mg, respectively) used in the preparation, and NC is the formulation that only used oleic acid in the preparation.

The graphs resulting from the two methods of analysis are shown in Figure 3S, with the observed differences being due to the characteristics of each method of analysis. Similar differences were obtained by Filipe *et al*.^30^, who used the dynamic light scattering (DLS) and NTA techniques to investigate mixtures of polystyrene particles with two different diameters.

Measurements of hydrodynamic diameter and polydispersity after 120 days showed that in most cases, the different amounts of neem oil in the nanoparticles did not substantially alter the stability of the system, with only the NC_10 nanoparticles presenting significant differences in size during the period. Some changes were noted in the average diameters of the nanoparticles, although these were not significant.

**Figure 1:**
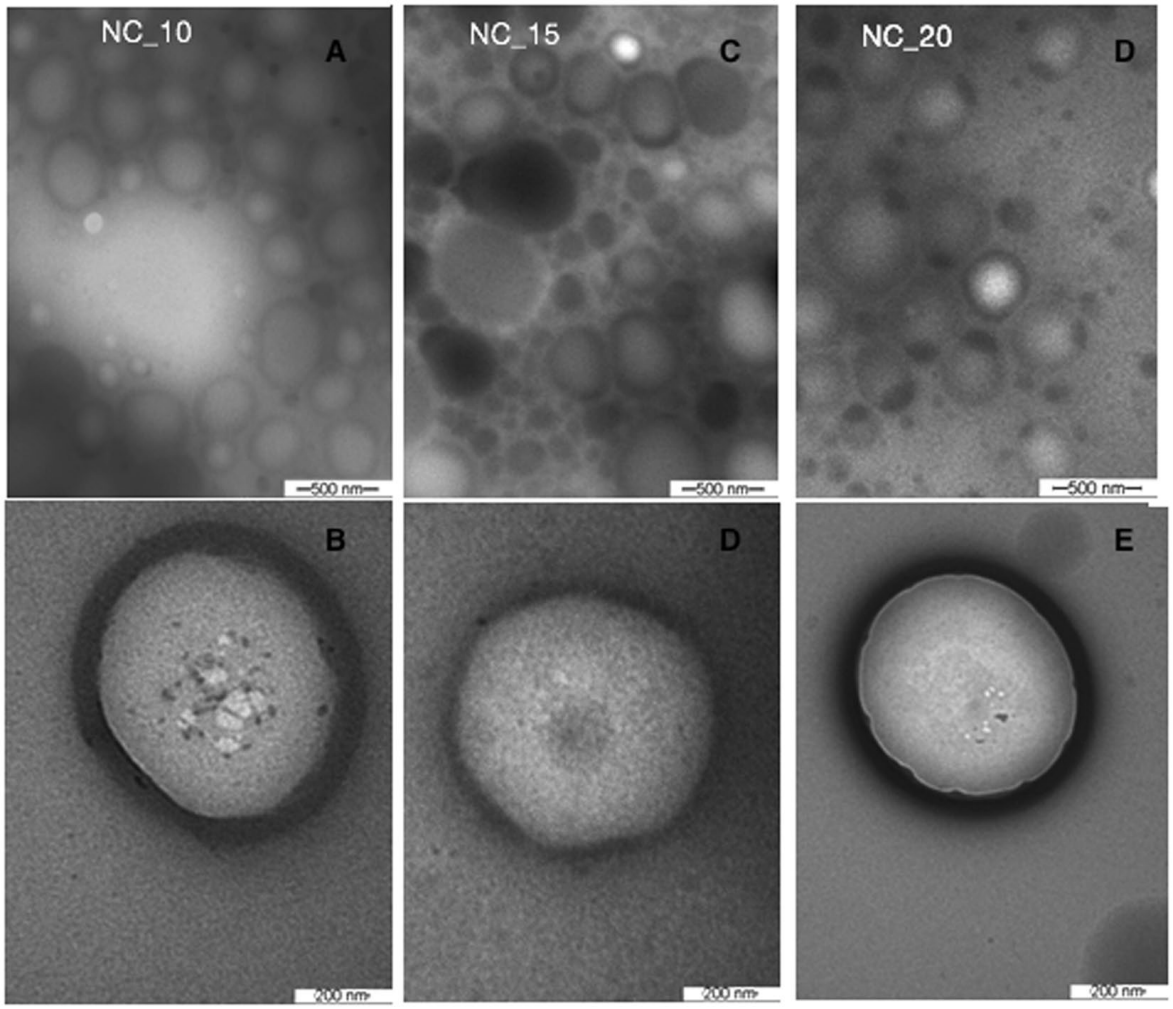
Transmission electron micrographs of nanocapsules containing neem oil. NC_10 (**A**,**B**), NC_15 (**C**,**D**), and NC_20 (**E**,**F**). Magnification 216000x in **A**,**C**,**E**; and 89230x in (**B**,**D**,**F**).

The nanocapsules were characterized by transmission electron microscopy immediately after synthesis. The micrographs obtained showed that an increase in the quantity of neem oil employed in the synthesis did not affect the morphology of the nanocapsules (Fig. 1). In all the formulations, the nanocapsules presented spherical morphology with diameters between 250 and 280 nm.

### Evaluation of cell viability

The assessment of cell viability was performed using the tetrazolium reduction technique with different cell lines (embryonic 3T3 fibroblasts, HeLa tumor cells, HaCat keratinocytes, and V79-4 pulmonary fibroblasts). The cell lines showed different responses when exposed to nanocapsules that did not contain neem oil (Figure 4S). These nanocapsules only contained oleic acid (200 mg), so the results were indicative of toxicity caused by the oleic acid. The greatest resistance was shown by the HeLa cells, which presented an IC_50_ of 4.75 mg/mL oleic acid, while the V79-4 cells were most sensitive, with IC_50_ of 0.12 mg/mL oleic acid. In previous work, no toxicity of PCL nanoparticles was found in the studies of Filipovic *et al*.^31^ and Campos *et al*.^32^, supporting the findings of the present work and suggesting that the toxicity was probably due to the oleic acid present in the formulation.

When the cell lines were exposed to the NC_20, NC_15, and NC_10 formulations, the greatest sensitivity was shown by the V79-4 cells (Figure 5S), with evidence for increasing viability, albeit slight, at higher concentrations of neem oil and lower concentrations of oleic acid.

The results showed that the formulation that presented the greatest cytotoxicity towards all the cell lines was NC_10. A possible explanation was the likely toxicity of oleic acid, used as a complement in the preparation of the NC_10 and NC_15 nanocapsules, which has been described previously in studies using blood cell lines^33–35^. Another contributing factor could have been increased viability in the presence of azadirachtin, the main compound present in neem oil, as found previously in viability assays employing mouse 3T6 fibroblast cells^36^.

Hence, it appears that in the case of the nanocapsules containing neem oil, a higher concentration of oleic acid and a lower concentration of azadirachtin could have resulted in higher toxicity.

The results for the HeLa and 3T3 cells showed that the former presented lower IC_50_ values and were more sensitive to the nanocapsules containing neem oil, compared to the 3T3 cells (Table 2). Similar results were obtained in viability tests performed by Ricci *et al*.^37^, where 3T6 fibroblast cells and HeLa tumor cells were exposed to a methanolic extract of neem oil without azadirachtin in its composition, with the tumor cells showing greater sensitivity to the extract, probably caused by alterations in the plasma membrane.

**Table 2:** IC_50_ values obtained for the different formulations and cell lines (3T3, HeLa, HaCat, and V79-4) using the MTT reduction assay.

Formulation	IC_50_ (mg/mL)			
	3T3	HeLa	HaCat	V79-4
NC_20	3.82	2.99	2.9	0.5
NC_15	3.43	3.71	3.28	0.29
NC_10	1.4	2.33	1.88	0.06
NC	3.55	4.75	3.46	0.12

The effect of the nanocapsules containing neem oil on the V79-4 cell line was similar to the behaviors observed for the other cell lines (with lowest IC_50_ for NC_10), although the raw values were much lower, reflecting greater sensitivity to the nanocapsules, with or without neem oil in their compositions. This could be explained by the different cell lines presenting different responses to the compounds. It should be noted that the HeLa and HaCat cells, which showed similar patterns of cytotoxicity, are human cell lines, while the 3T3 and V79-4 cells, which also showed similar patterns of cytotoxicity, are rodent cell lines.

The neem oil used to prepare the nanocapsules contains over 300 secondary metabolites, with azadirachtin being the predominant compound. It is therefore likely that the IC_50_ values obtained here for the NC_15 and NC_10 nanocapsules, as well as the results reported in the literature, were influenced not only by azadirachtin, but also by its interaction with the other active components of neem oil and the oleic acid used in the preparation of the nanocapsules. In the case of the NC_20 nanocapsules, which did not contain oleic acid, there was only interaction between azadirachtin and the other metabolites present in neem oil.

The greater viability of the cells exposed to the NC_20 nanocapsules could be explained by a possible contribution of azadirachtin to cell proliferation. Even if the other metabolites presented cytotoxic activity, azadirachtin compensated the negative effects on viability by inducing the division of unaffected cells. Considering the possible toxicity of oleic acid and the secondary metabolites present in neem oil, their combined effects could provide a reason for the very low IC_50_ values found for the NC_10 nanocapsules, because this formulation contained the greatest amount of oleic acid and the smallest amount of azadirachtin, leading to high cytotoxic activity.

### Comet assays

**Figure 2:**
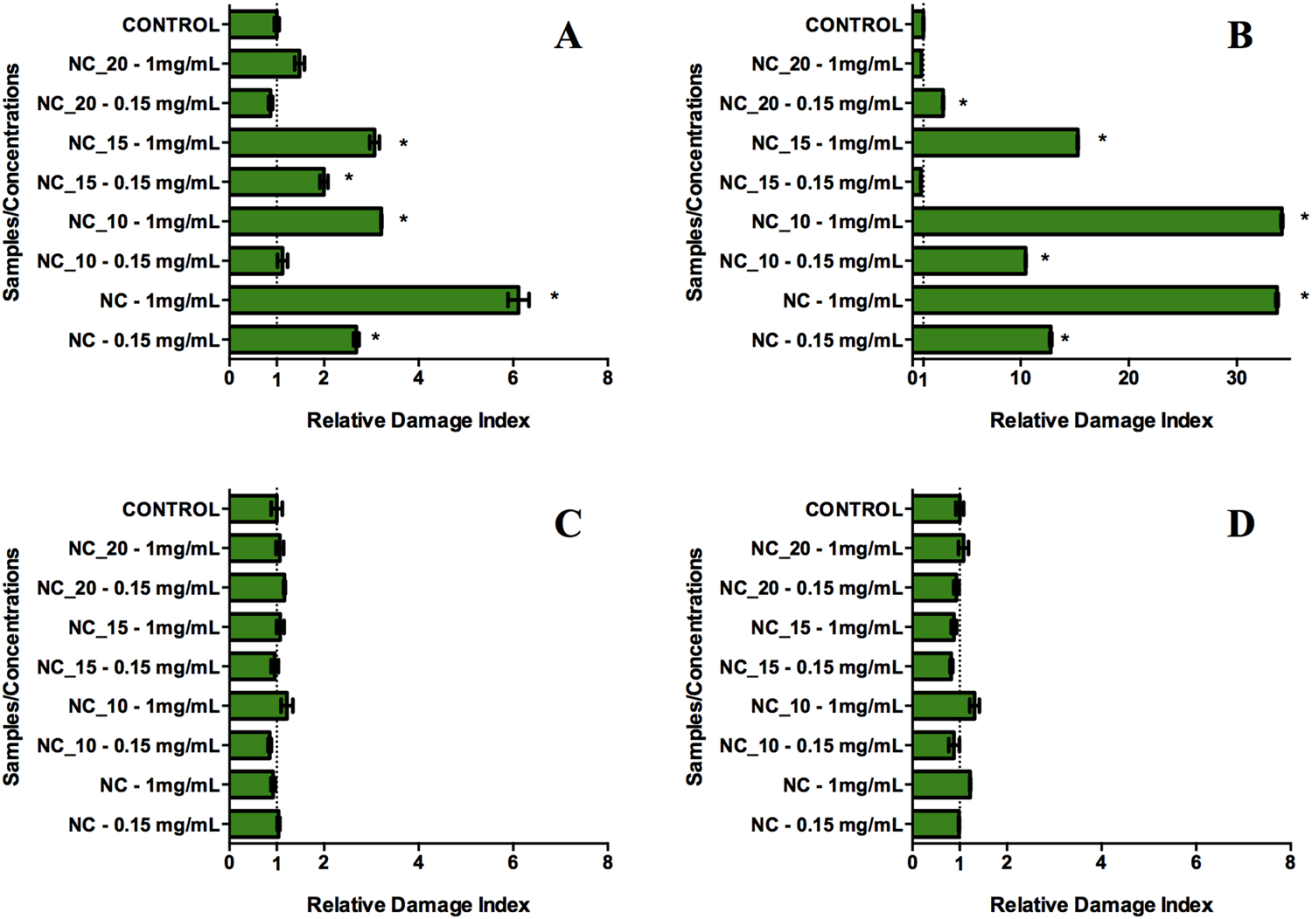
Evaluation of genotoxicity of the NC_20, NC_15, NC_10, and NC formulations using the comet assay with different cell lines. (**A**) 3T3; (**B**) HeLa (note the higher values of the x-axis, compared to the other graphs); (**C**) HaCat; D: V79-4.

The results showed that the HeLa cells presented the greatest sensitivity to the formulations (Fig. 2). The HaCat and V79-4 cells exposed to the formulations showed no significant differences in DNA damage, compared to the control. The 3T3 cells showed greater damage when exposed to the nanocapsules containing only oleic acid, and similar behavior was observed for the HeLa cells. The 3T3 and HeLa cells exposed to the NC_20 treatment showed no significant damage, compared to the control.

In the treatments using nanocapsules containing neem oil, the damage index values were inversely proportional to the amount of oil used in the formulation, with the NC_10 formulation at the highest concentration (1 mg/mL) causing the greatest DNA damage in the cells, and the HeLa cells being the most affected. In similar work by Mosesso *et al*.^38^, no genotoxicity was observed for azadirachtin.

The findings demonstrated that the different toxicities exhibited by the various formulations could be explained by the interactions among the components of the neem oil. In the case of the NC_15 and NC_10 formulations, the relative damage index (RDI) was much higher at the highest concentration tested, for the two cell lines that showed significance (3T3 and HeLa). This was not observed in the case of the NC_20 formulation, for any of the cell lines. A possible reason for this difference was the presence of oleic acid in the formulations. Hence, in the case of the NC_15 and NC_10 formulations, the differences observed for the concentrations tested was due to the interaction of oleic acid with secondary metabolites present in the neem oil. This feature was not observed for the NC_20 formulation, because it did not contain oleic acid.

### Allium cepa chromosomal aberration assays

**Figure 3:**
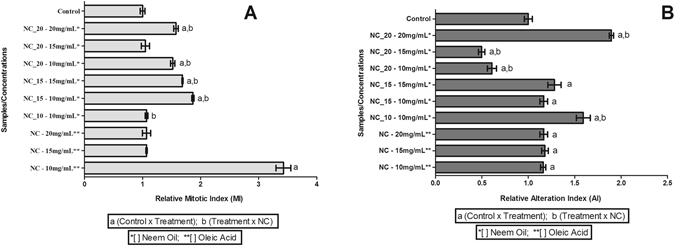
Evaluation of genotoxicity of the NC_20, NC_15, NC_10, and NC formulations using *Allium cepa*. (**A**) Relative mitotic index; (**B**) Relative abnormality index. ANOVA-Tukey statistical analysis, p < 0.05 (a − control × treatment; b − treatment × nanocapsules). Data presented as mean ± SE (n = 9).

The *Allium cepa* test was used to determine the mitotic index and abnormality index values in the presence of the PCL nanocapsules, with or without neem oil. With the exceptions of the NC_20 formulation at a concentration of 15 mg/mL and the NC_10 formulation at a concentration of 10 mg/mL, there were significant increases, compared to the negative control, after exposure for 24 h (Fig. 3A).

The abnormality index values were significantly higher, relative to the negative control, for the following formulations: NC_20 at 20 mg/mL, NC_15 at concentrations of 15 mg/mL and 10 mg/mL, and NC_10 at 10 mg/mL. The NC_20 formulations at concentrations of 15 mg/mL and 10 mg/mL showed significantly lower AI values, compared to the negative control (Fig. 3B).

The mitotic index value obtained for the NC formulation prepared with 10 mg/mL of oleic acid was substantially higher than the values obtained for the three nanocapsule formulations containing neem oil at a concentration of 10 mg/mL. This could have been due to the inhibition of cell proliferation by a component of neem oil (nimbolide), as described by Kumar *et al*.^39^ and Roy *et al*.^40^. Elsewhere, Pereira *et al*.^41^ and Grillo *et al*.^42^ reported slightly higher mitotic index values in the presence of nanoparticles without the bioactive compound, suggesting that PCL might act to stimulate cell division.

Comparison of the activity of the three formulations containing neem oil at 10 mg/mL with the formulation without neem oil (NC, with 10 mg/mL of oleic acid) revealed a higher AI for treatment with NC_10, compared to NC. The AI values increased in the order NC_20 < NC_15 < NC_10, and the AI value obtained for NC_20 was significantly lower, compared to NC. Additionally, the highest AI value was obtained for the treatment with NC_20 at the highest concentration (20 mg/mL), similar to the findings of Kwankua *et al*.^43^.

### Determination of minimum inhibitory concentration

The microplate assays enabled identification of the lowest concentration of each formulation capable of inhibiting the growth of three types of microorganisms: *Escherichia coli*, *Candida albicans*, and strains of bacteria isolated from soil (Table 3).

**Table 3:** Minimum inhibitory concentrations (MIC) of the NC_20, NC_15, NC_10, and NC formulations towards three types of microorganisms: *E*. *coli*, *C*. *albicans*, and bacteria isolated from soil.

Microorganisms	NC_20 (mg/mL)	NC_15 (mg/mL)	NC_10 (mg/mL)	NC (mg/mL)
Bacteria isolated from soil	5	2.25	1.5	2
*Escherichia coli*	2	3	0.5	2
*Candida albicans*	2	0.75	0.5	2

The formulation with the lowest concentrations able to inhibit the growth of the microorganisms was NC_10 (Table 3). Most of the formulations showed higher minimum inhibitory concentration values for the bacteria isolated from soil, which could be explained by the coexistence in the soil of different strains of bacteria with differing degrees of resistance, resulting in the requirement for a greater quantity of active agent in order to inhibit growth.

### Molecular analysis of the soil microbiota

**Figure 4:**
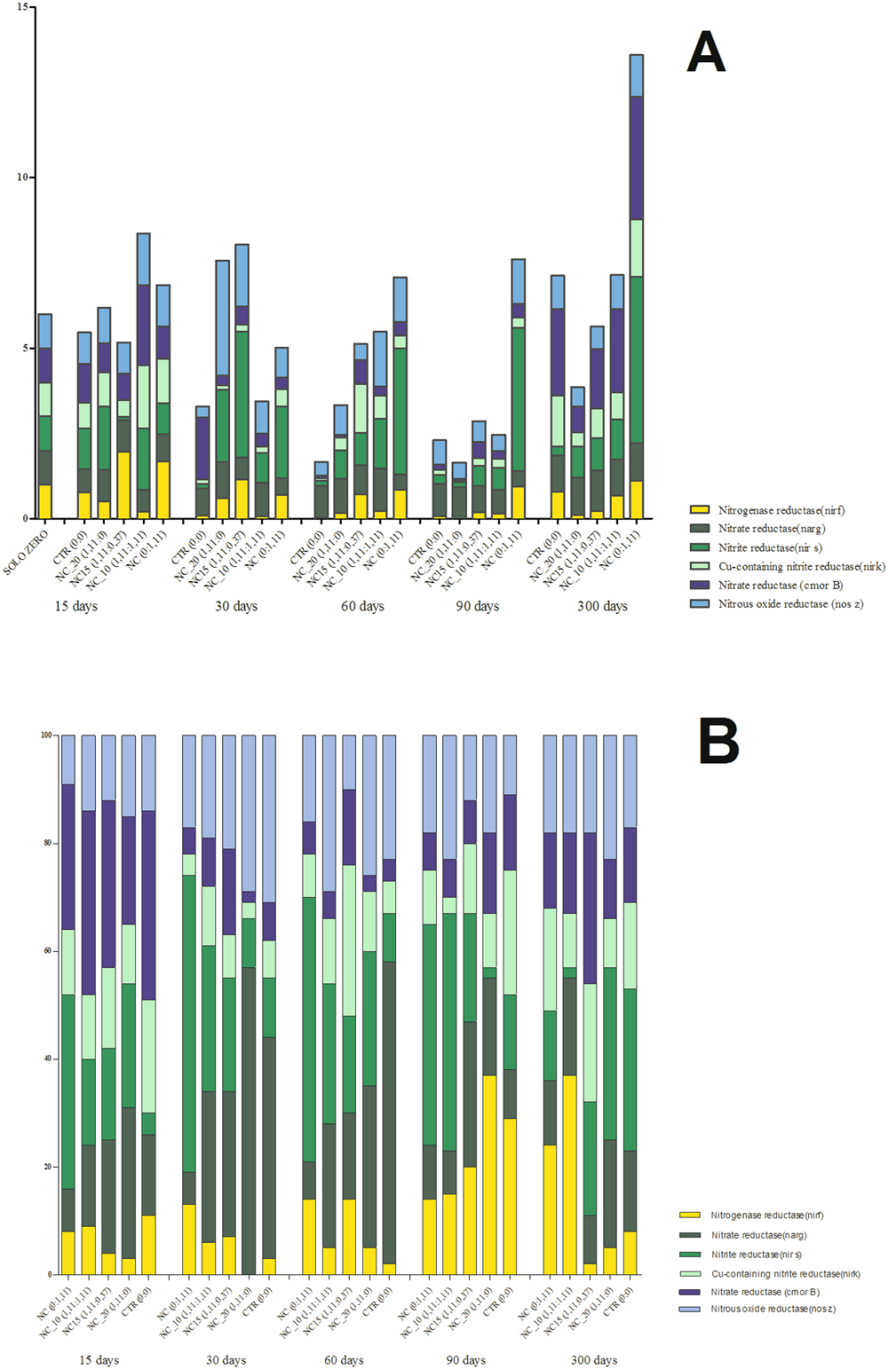
Molecular analysis of the soil nitrogen cycle bacteria involved in the phases of fixation and denitrification, after treatment with the NC_20, NC_15, NC_10, and NC formulations. (**A**) Relative quantification of genes by qPCR; (**B**) Percentage distribution of each gene. The analyses were performed by qPCR of the following genes: *nifH*, *narG*, *nirS*, *nirK*, *norB*, and *nosZ*, at 15, 30, 60, 90, and 300 days after the first applications of the formulations.

The results for the soil microbiota indicated that the negative control presented cyclic behavior, with the amount progressively decreasing for the first three extractions (after 15, 30, and 60 days), then increasing after 90 days (Fig. 4A). This could have been due to the physical conditions (such as temperature and humidity) to which the soils were exposed (although it should be noted that the temperature was controlled during the experiment). After 15 days, there was an increase of the soil microbiota exposed to NC (0:0.12) and a decrease of the soil microbiota exposed to NC_20 (0.12:0), compared to the control (Fig. 4A). The distribution of the different types of bacteria was similar to that of the control (Fig. 4B).

After 30 days and two applications of the formulations, the microbiota showed greater similarity for the different treatments, although the NC treatment (0:0.12) resulted in a greater quantity of microorganisms, compared to the other samples tested. The distributions of the microbiota in the soils treated with the active formulations showed greater differences, relative to the control, with a decrease of the microorganisms responsible for nitrogen fixation in most of the soils.

After 60 days, there appeared to be a recovery of the microbiota in the soils treated with the nanoparticles (Fig. 4A), with the soil treated using the NC formulation (0:0.12) still showing the highest levels. The treatment with NC_20 showed a smaller quantity of bacteria up to 60 days after the first exposure, followed by increases at 90 and 300 days, although after 60 days there was a small increase in nitrogen-fixing bacteria. The negative control showed the highest proportion of denitrifying bacteria, especially the phase 1 organisms (Fig. 4B).

At 90 days after the first exposure, the numbers of bacteria increased in the control soil and the soils treated with NC_15, and NC_20. In terms of the distribution of bacteria (Fig. 4B), the soils treated with NC (0:0.12) and NC_10 showed the least similarity to the control.

At 300 days after the first exposure, the different treatments showed similar numbers of organisms to each other and to the control. However, the distribution of the different types of organisms varied according to the treatment, with the greatest differences, relative to the control, for the treatments using formulations containing higher amounts of oleic acid and the treatment using equal proportions of oleic acid and neem oil. The control and the various treatments also showed increases in nitrogen fixing bacteria, suggesting recovery of the soils after 300 days.

In terms of the quantity of nitrogen fixing bacteria, the most obvious feature was their disappearance in the soil sample exposed to nanocapsules containing only neem oil (NC_20, 0.12:0) within 90 days after the first application. This indicates that there was lower nitrogen fixation in the soil exposed to NC_20. This could have been due to the fact that neem oil inhibits nitrification^44^, hence increasing the amount of ammoniacal nitrogen and raising the pH of the soil, which can affect the nitrogen fixing bacteria.

In the case of the bacteria responsible for the first stage of denitrification, the most significant result was the decrease in the number of these bacteria in the soil exposed to NC (0:0.12) at 300 days after the first exposure. For the bacteria of the second stage of denitrification, the most important effect was the decrease of the *cnorB* gene in the soil treated with NC_20 (0.12:0) for all the periods analyzed, relative to the negative control.

Analysis of the genes of soil bacteria in order to evaluate the impacts of new compounds used in agriculture is a technique that is still in its early development. The results obtained to date provide the basis for further investigations, and it is important to note that much work is still needed to identify end-points that accurately reflect the action of these compounds on the microbiota.

### Phytotoxicity assay

**Figure 5:**
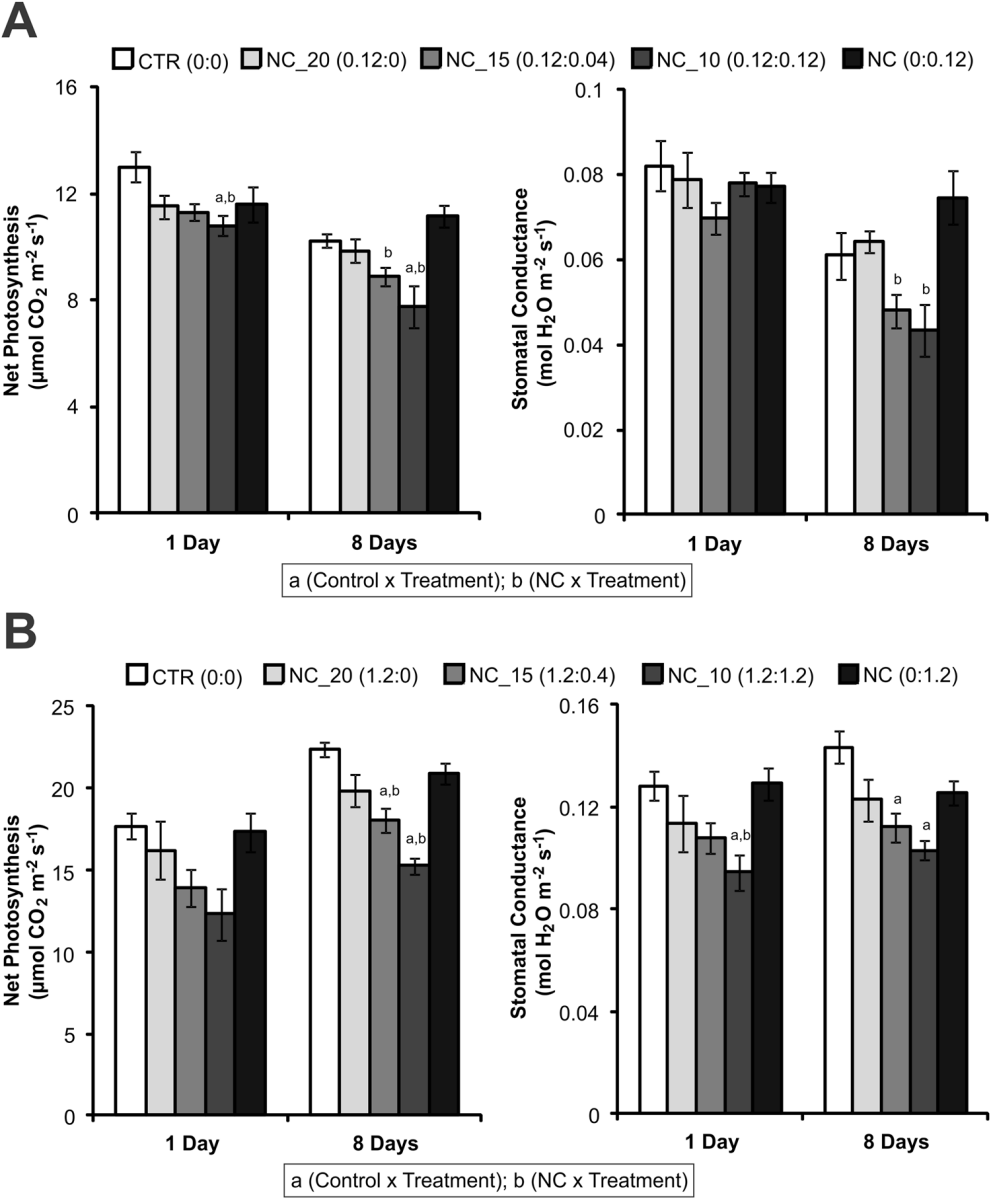
Net photosynthesis and stomatal conductance of maize plants 1 and 8 days after treatment with the NC_20, NC_15, NC_10, and NC formulations, or with water (control). The nanoformulations were applied at (**A**) 0.12 or (**B**) 1.2 mg/m^2^. Data presented as mean ± SE (n = 9).

At the neem oil concentration indicated for field applications (0.12 mg/m^2^), after one and eight days of exposure to the formulations, there was a reduction in the net photosynthetic CO_2_ assimilation rate of the maize plants only in the case of the NC_10 treatment, compared to the control and NC treatments (Fig. 5A). After eight days of exposure, there was also a decrease of this parameter for the NC_15 treatment, compared to the NC treatment. The stomatal conductance of the maize plants only decreased after eight days for the NC_15 and NC_10 treatments, compared to the NC treatment. When a ten-fold higher neem oil concentration was applied (1.2 mg/m^2^), negative effects on the net photosynthesis of the maize plants were observed eight days after exposure to NC_15 and NC_10, compared to the control and NC (Fig. 5B). Stomatal conductance was reduced on both days for the NC_10 treatment, compared to the control, and for the NC_15 treatment eight days after exposure, compared to the control. The application of NC_20 at both 0.12 and 1.2 mg/m^2^ dosages did not affect the analyzed physiological parameters of the maize plants (Fig. 5). Additionally, no effects were observed after treatment with NC, compared to the control (Fig. 5), in agreement with previous studies that showed that unloaded PCL nanocapsules did not affect the physiological parameters of maize and mustard plants^45, 46^. The absence of an effect of the nanoformulations without neem oil, used as negative controls, indicates that the inert materials used in the encapsulation process did not cause adverse effects^47^.

It is notable that when there was only neem oil or oleic acid in the nanocapsules, they did not impair gas exchange of the maize leaves. However, when there was a higher oleic acid concentration together with neem oil in the nanocapsules, they became phytotoxic, as observed for the NC_10 and, at lower intensity, the NC_15 treatments. The results highlight the negative impact of neem oil mixed with oleic acid in the nanocapsules on the gas exchange parameters of plants, as observed in the cell viability, microorganism, and genotoxicity assays. Although macroscopic symptoms were not observed in the leaves until eight days after treatment with the formulations (data not shown), the reduction of net photosynthesis and stomatal conductance rates by the NC_10 and NC_15 formulations would probably be reflected in long-term undesirable effects in maize plants, such as reduced biomass and yield. The relevance of these results is increased by the fact that very few studies have evaluated the effects of polymeric nanoparticles on plants^48, 49^. Even in studies with metallic nanoparticles, which are more common, contrasting results regarding the effects of these nanomaterials on photosynthesis have been obtained^50^.

## Conclusions

Although nanotechnology is still at an early stage in the agricultural sector, it is clear that there is growing interest in its use. The results obtained in this study showed that PCL nanocapsules loaded with neem oil presented good colloidal stability and that all the nanoparticles were spherical. The cyto-, geno-, and phytotoxicity studies showed that the NC_10 formulation (100 mg of neem oil and 100 mg of oleic acid) presented the highest toxicity against nontarget organisms, while the NC_20 formulation (200 mg of neem oil) presented the lowest toxic effect. These results may have been due to the interaction of components of neem oil with the oleic acid used in some formulations.

The results demonstrate the importance of studying the toxicity of nanopesticides as a key factor in selection of the best formulations for use in agricultural applications. Further studies will be performed to evaluate the biological activity of the formulation with lowest toxicity and to understand the mechanism of action of this nanopesticide in target organisms.

## Materials and Methods

### Preparation of PCL nanocapsules

The polymeric nanocapsules were prepared using the oil in water emulsion and solvent evaporation method. Different proportions of neem oil and oleic acid (1:1, 1.5:0.5, and 2:0, w/w) were dissolved in 10 mL of acetone. This solution was added to 20 mL of chloroform containing 400 mg of PCL polymer (80,000 g/mol) and the mixture was sonicated for 1 min at 100 W. The resulting pre-emulsion was added to an aqueous solution containing 150 mg of polyvinyl alcohol (PVA) surfactant and then sonicated for 8 min to form the emulsion. The solvent was eliminated using a rotary evaporator at 40 °C and the emulsion was made up to a volume of 10 mL.

PCL nanocapsules were also prepared without neem oil, using the same technique but only dissolving oleic acid in the acetone. For better clarity, the quantities of neem oil and oleic acid used in preparing the formulations are provided in Table 1S.

### Characterization

#### Size distribution, polydispersity index, and zeta potential

The dynamic light scattering (DLS) and nanoparticle tracking analysis (NTA) techniques were used to measure the average diameter (hydrodynamic diameter) of the nanocapsules according to time (up to 120 days). For the DLS measurements, nanocapsules with or without neem oil were diluted in deionized water (1:1000, v/v) and measured using a Zetasizer ZS90 system (Malvern Instruments, UK) at 25 °C and with a fixed angle of 90°. The results were expressed as the mean of three determinations. For the NTA technique, suspensions of nanoparticles with or without neem oil were diluted 5000 times and analyzed using a NanoSight LM10 instrument equipped with a 532 nm laser, a CMOS camera, and NanoSight v. 2.3 software. The zeta potential of the nanocapsules was determined by the microelectrophoresis technique, also using the Zetasizer ZS90 instrument. All the measurements were performed in triplicate and the results were expressed as means and standard deviations.

#### Transmission electron microscopy (TEM)

The morphology of the nanoparticles was investigated immediately after preparation, using a Zeiss LEO 906 microscope operated at 80 kV, at the Biology Institute of UNICAMP. Drops of the nanoparticle suspensions, contrasted with uranyl acetate, were placed on 200–300 mesh grids coated with Formar (a low absorption resin). The grids were analyzed after being allowed to dry by evaporation^51^.

### Toxicity analyses

#### Samples and concentrations

For the toxicity analyses, the three formulations of PCL nanocapsules containing neem oil were labeled according to the quantity of neem oil used in the preparation (NC_20, NC_15, and NC_10), as shown in Table 1S. The concentrations were calculated according to the quantity of neem oil used in the preparation, per mL of the final formulation. For example: NC_20 – 1 mg/mL, where NC_20 is the formulation for which 200 mg of neem oil was used in the preparation, and 1 mg/mL is the theoretical concentration of neem oil used in the tests.

The PCL nanocapsules without neem oil were labeled NC, and the concentrations were calculated based on the amount of oleic acid used in the preparation (for formation of the oily core), per mL of the final formulation. For example: NC – 1 mg/mL, where NC is the formulation for which 200 mg of oleic acid was used in the preparation, and 1 mg/mL is the theoretical concentration of oleic acid used in the tests.

For evaluation of the action of the nanocapsules on the soil bacteria and the maize plants, labeling was based on the concentration (mg/m^2^) of neem oil indicated for field applications (0.12 mg/m^2^), together with the concentration of oleic acid present in tests using the NC_15, NC_10, and NC formulations. For example, NC_15 (x:y), where NC_15 is the formulation applied and x and y are the theoretical concentrations (in mg/m^2^) of the neem oil and oleic acid, respectively, employed in the assays.

#### Cell mitochondrial viability assays

Cell mitochondrial viability was determined using the tetrazolium bromide reduction technique (MTT assay). The procedure started with the plating out of a cell suspension containing 0.5 × 10^5^ cells/mL. The cell lines used were 3T3 (albino Swiss mouse), HeLa, V79-4, and HaCat. After 24 h (the time required for adherence and stability), the cells were placed in contact with the NC_20, NC_15, and NC_10 samples containing neem oil at concentrations in the ranges 0.2–7, 0.15–5.25, and 0.1–3.5 mg/mL, respectively, and with NC samples containing oleic acid at concentrations in the range 0.2–7 mg/mL. Incubation was continued for another 24 h, followed by washing the wells with phosphate buffered saline (PBS). Addition was then made of 100 μL of 0.5 mg/mL tetrazolium bromide solution and the plates were left in the incubator for 3 h. The formazan solution was then discarded, DMSO was added, and measurements were made at 540 nm using a plate reader (Readwell Plate, Robonik). All the tests were performed in sextuplicate for each concentration of each formulation tested.

The results were analyzed considering 100% viability for the mean absorbance obtained for the untreated control. The cell viability at each concentration could then be calculated relative to the negative control.

#### Comet assays and Allium cepa chromosomal aberration tests

The comet assays were performed using cell lines 3T3 (albino Swiss mouse), HeLa, V79-4, and HaCat, which were placed in contact with the NC_20, NC_15, NC_10, and NC formulations at concentrations of 0.15 and 1 mg/mL for 1 h, then mixed with 0.5% low melting agarose and distributed on glass slides that had been previously prepared with a layer of 1.5% standard agarose (normal melting point). Duplicates were determined for each concentration of each treatment, as well as the negative control. After preparation, the slides were immersed in lysis solution, where they remained for approximately 1 h. At the end of the lysis period, the slides were washed in neutralization solution for 5 min and placed in an electrophoresis cell containing buffer at 4 °C, where they were allowed to rest for 20 min, followed by initiation of the run, which lasted another 20 min (at 30 V, 300 mA, and 10 W). Immediately after the run, the slides were washed in neutralization solution for 5 min and allowed to dry overnight at ambient temperature.

For staining of the slides with silver, they were first washed with distilled water and dried in an oven at 37 °C for 2 h. They were then placed in fixing solution for 10 min, washed again with distilled water, and allowed to dry overnight at ambient temperature. Staining was performed with silver solution for 35 min, under agitation. At the end of this process, the slides were immersed in stop solution for 5 min, washed with distilled water, and dried at ambient temperature.

The slides obtained from the comet assays were analyzed using an optical microscope, averaging 100 cells for each treatment. Damage was rated using a scale from 0 (no DNA breaks, no visible tail) to 4 (greatest quantity of DNA breaks, largest tail) and the scores and damage index values were calculated (Table 2S).

For the chromosomal aberration assays, *Allium cepa* seeds were germinated on filters moistened with water, in Petri dishes, for about 4–5 days. When the roots reached lengths between 1 and 2 cm, they were placed in contact for 24 h with samples NC_20 (concentrations of 20, 15, and 10 mg/mL), NC_15 (concentrations of 15 and 10 mg/mL), NC_10 (concentration of 10 mg/mL), and NC (concentrations of 20, 15, and 10 mg/mL). After the contact period, the roots were washed with distilled water, immersed in Carnoy fixing solution (ethanol and acetic acid at a ratio of 3:1, v/v), and stored in a refrigerator.

The roots were prepared by first washing with distilled water, followed by acid hydrolysis for 9 min using 1 mol/L hydrochloric acid, at a constant temperature of 60 °C. The roots were again washed with distilled water, followed by staining with Schiff reagent for 2 h. For subsequent analysis using an optical microscope, slides were prepared in triplicate, with crushing of the meristematic region in a drop of 2% acetic carmine.

Cytotoxicity and genotoxicity evaluations were performed using analysis of around 500 cells, with an average of 1,500 cells per sample for each concentration tested. The results were used to calculate the mitotic index (MI) and abnormality index (AI) values for each treatment, as well as for the negative blank, as shown in Scheme 1.

Statistical analysis of the results of the two assays employed ANOVA followed by Tukey’s HSD post-hoc test for equal variances. GraphPad Prism software was used, and the significance level adopted was p < 0.05.

#### Minimum inhibitory concentration (MIC)

Minimum inhibitory concentrations were determined using strains of *Escherichia coli* (ATCC 25922) and *Candida albicans* (ATCC 10231), as well as bacteria isolated from soil.

For the isolation of bacteria from soil, a mixture of 1 g of soil and 9.5 mL of 0.1% sodium pyrophosphate solution was maintained under agitation for 30 min. The resulting solution was diluted with 0.9% saline (1:10, v/v), inoculated (1 mL) into Petri dishes containing Tryptic Soy Agar culture medium, and incubated at 37 °C for 48 h. Samples were then collected from the colonies formed and frozen in Brucella broth (a medium suitable for freezing), consisting of enzymatic casein hydrolysate, peptic digest of animal tissue, yeast extract, dextrose, sodium chloride, and sodium bisulfite.

The microorganisms (*E*. *coli*, *C*. *albicans*, and soil bacteria, frozen at −80 °C) were cultivated in test tubes containing Müeller Hinton culture broth that had been previously autoclaved and cooled before adding the microorganisms. Small amounts of the thawed samples were transferred to the tubes using a nickel/chromium loop and incubated at between 35 and 37 °C for 24 h. Counting was then performed using 0.4% Trypan Blue in a Neubauer chamber. If necessary, the samples were diluted in culture broth to achieve a concentration of 5 × 10^6^ CFU/mL.

Using a 96-well microplate, serial dilutions in Müeller Hinton culture broth were made of the samples NC (0.1–1 mg/mL), NC_10 (0.05–0.5 mg/mL), NC_15 (0.075–0.75 mg/mL), and NC_20 (0.1–1 mg/mL). Subsequently, 10 μL aliquots of the suspensions containing the microorganisms were added to the wells and the plate was incubated at 35 °C for 24 h. All the tests were performed in duplicate for each sample.

After the initial incubation period, addition was made of 10 μL aliquots of Resazurin growth indicator solution (6.75 mg/mL/well) and the plate was kept at 35 °C for a further 24 h. Each plate included a positive control (without inclusion of any type of test sample) and a contamination control (without the addition of microorganisms).

After 24 h, a change in color in the wells from dark blue to pink indicated growth of the microorganisms, while growth was inhibited in wells that retained a blue color. After completion of the test, inhibition of growth was confirmed by plating the contents of the wells on culture medium and observing the occurrence of growth of the microorganisms.

#### Molecular analysis of the soil microbiota

The soil used in the tests contained 14% organic matter and had a pH of 6.80. The soil was sieved through a plastic sieve and kept moist in a heated cabinet at 25 °C for 15 days. After this period, the DNA was extracted from a sample of the soil (denoted soil zero), followed by separation of the soil into 5 portions of 30 g, to which the nanocapsules (NC_20, NC_15, NC_10, and NC) were added using sprays. The applications of the three formulations containing neem oil were based on the dosage of the oil applied in the field (0.12 mg/m^2^). The formulation that did not contain neem oil (NC) was applied to the soil at an oleic acid concentration equivalent to 0.12 mg/m^2^. After 15 days, further applications of the nanocapsules were made, using the same concentrations.

Extraction of the soil bacteria DNA employed the PowerSoil DNA Isolation Kit (MO BIO Laboratories, Inc.). Six extractions were performed: a control extraction before the start of the trial (soil zero), an extraction 15 days after the first application, and four extractions after the two exposures (at 30, 60, 90, and 300 days).

Following extraction, the DNA samples were quantified using a Qubit dsDNA BR Assay Kit and a Qubit 2.0 fluorometer (Invitrogen). All the samples were then diluted to a concentration of 100 ng/mL for use in the real-time polymerase chain reaction.

Real-time PCR was used to monitor the behavior of the bacterial communities in soil treated with the PCL nanocapsules containing neem oil. The reactions (individually for each primer) were performed using a final volume of 25 μL, with 12.5 μL of Planium SYBR Green qPCR SuperMix-UDG with ROX (Invitrogen), 1 μL of each primer (sense and antisense), and 1 μL of the sample of DNA extracted from the soil. The volume was completed using ultra-pure autoclaved water.

The reactions were conducted in a StepOne thermocycler (Applied Biosystems), using the following amplification conditions (adapted from Jung *et al*., 2011): 3 min at 95 °C for the initial denaturation, then 40 cycles of 95 °C for 45 s, 60 °C for 45 s, and 72 °C for 45 s.

The fluorescence emitted by the SYBR Green was measured at the end of each incubation step at 72 °C, and in the exponential phase of the amplification reached a threshold value denoted Ct (the cycle time, namely the time required for the fluorescence to reach the threshold for emission in the exponential phase)^52^. The results were analyzed using relative quantification, with calculation of ΔCt employing 16S rRNA as the reference gene and the initial zero soil as the reference sample. The calculations were performed using the StepOne Plus software of the equipment.

#### Leaf gas exchange measurements

Phytotoxicity assays were carried out by analyzing leaf gas exchange parameters of maize plants (*Zea mays* L., hybrid SHS 3031). The whole experiment was performed in a greenhouse under natural conditions of light and temperature. The seeds were sown in plastic pots filled with 0.8 L of a mixture of clay soil, sand, and cattle manure (2:2:1). After four weeks of germination, the maize plants were sprayed with water (control) or with the formulations. The nanocapsules containing neem oil (NC_20, NC_15, and NC_10) were applied at 0.12 mg/m^2^ (the dosage of the oil applied in the field) and 1.2 mg/m^2^. Similarly, the formulation that did not contain neem oil (NC) was applied at oleic acid concentrations equivalent to 0.12 and 1.2 mg/m^2^. Leaf gas exchange parameters (net photosynthesis and stomatal conductance) of nine plants were measured one and eight days after treatment applications, between 08:00 and 10:00 am, using a portable photosynthesis system (LI-6400XT, LI-COR Biosciences, Lincoln, NE, USA). The infrared gas analyzer (IRGA) was connected to the 6400-02B measuring chamber, where the leaves were exposed to saturating PAR (1,500 µmol m^−2^ s^−1^) and a flow rate of 400 mL min^−1^. The data were analyzed by ANOVA followed by Tukey’s HSD post-hoc test (p < 0.05), using the Statistica 10.0 software.

## Electronic supplementary material

Supplementary info
