# Supplementary material for: Nanocapsules Containing Neem (Azadirachta Indica) Oil: Development, Characterization, And Toxicity Evaluation
Source: Sci Rep. 2017 Jul 19;7:5929. doi: 10.1038/s41598-017-06092-4 (PMC5517525; doi:10.1038/s41598-017-06092-4)

NANOCAPSULES CONTAINING NEEM (*Azadirachta indica*) OIL: DEVELOPMENT, CHARACTERIZATION AND TOXICITY EVALUATION.

Tatiane Pasquoto-Stigliani[1,4], Estefânia V. R. Campos[2], Jhones L. Oliveira[2], Camila M. G. Silva[3], Nathalia Bilesky-José[4], Mariana Guilger[1,4], Johann Troost[5], Halley C. Oliveira[5], Renata Stolf-Moreira[5], Leonardo F. Fraceto[2], Renata de Lima[1,4*]

[1] Programa de Pós-Graduação em Biotecnologia e Monitoramento Ambiental, Universidade de São Carlos (UFSCar), Sorocaba, SP, Brazil

[2] Universidade Estadual Paulista (UNESP), Instituto de Ciência e Tecnologia, Departamento de Engenharia Ambiental, Sorocaba, SP, Brazil

[3] Departamento de Bioquímica, Universidade Estadual de Campinas (UNICAMP), Campinas, SP, Brazil

[4] Laboratório de Biotecnologia, Universidade de Sorocaba (UNISO), Sorocaba, SP, Brazil

[5] Departamento de Biologia Animal e Vegetal, Universidade Estadual de Londrina (UEL), Londrina, PR, Brazil

* Corresponding author (R. Lima). Tel.: 55 (15) 2101-7000; Fax: 55 (15) 2101-7000;

E-mail: renata.lima@prof.uniso.br

Table 1S: Oleic Acid and neem oil quantities used for nanocapsules preparation, final quantity of oily phase, "neem oil: oleic acid" mass proportion on oily phase and final concentration of azadirachtin present on each one of the formulations according to information provided by Neem oil manufacturer.

Formulations	Neem Oil	Oleic Acid	Oily phase	Proportion w/w	Azadirachtin
**NC_20**	200 mg	0 mg	200 mg	2:0	27,6 µg/mL
**NC_15**	150 mg	50 mg	200 mg	1,5:0,5	20,7 µg/mL
**NC_10**	100 mg	100 mg	200 mg	1:1	13,8 µg/mL
**NC**	0 mg	200 mg	200 mg	0:2	0 µg/mL

Table 2S: Equations used on comet assay and *Allium cepa* assay to obtain the desired parameters: Damage Index (DI) for comet assay; Mitotic Index (MI) and Alteration Index (AI) for *A. cepa* assay.

USED EQUATIONS		
Comet assay	$Score = \sum_{i=0}^{4} i \times Ni$	$DI = \frac{score}{total\ cells\ counted}$
Allium cepa assay	$MI = \frac{Total\ dividing\ cells}{Total\ cells\ counted}$	$MI\ rel. = \frac{IM\ of\ each\ treatment}{IM\ of\ controll}$
	$AI = \frac{Total\ of\ alterations}{Total\ dividing\ cells}$	$AI\ rel. = \frac{IA\ of\ each\ treatment}{IA\ of\ controll}$

Figure 3S: Initial size distributions of the nanocapsules with and without neem oil, using NTA (black lines) and DLS (red lines). A) NC; B) NC_10; C) NC_15; D) NC_20. The samples were diluted 5,000 and 1,000 times for the NTA and DLS analyses, respectively.

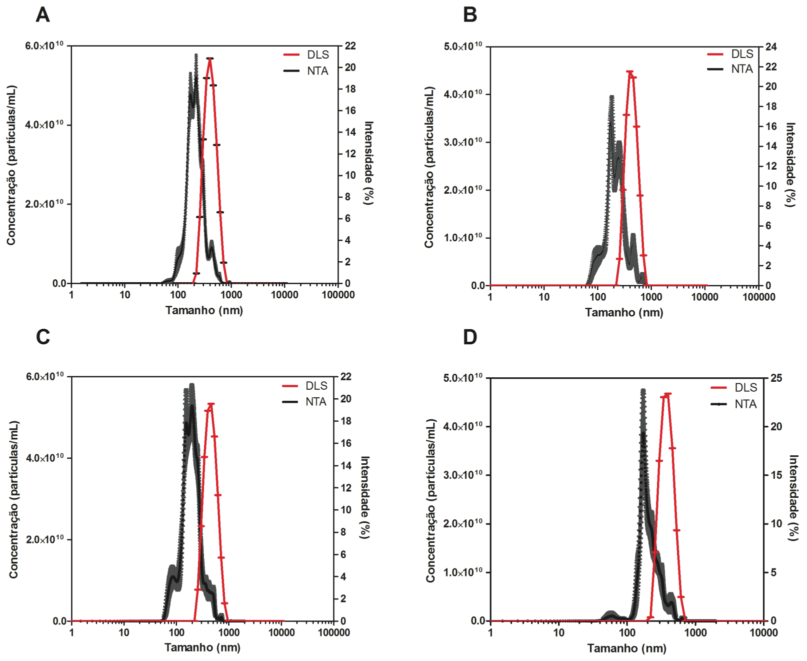

Figure 4S: Evaluation of cell viability using the MTT test applied to the NC formulation (without neem oil in its composition) and the 3T3, HeLa, HaCaT, and V79-4 cell lines.

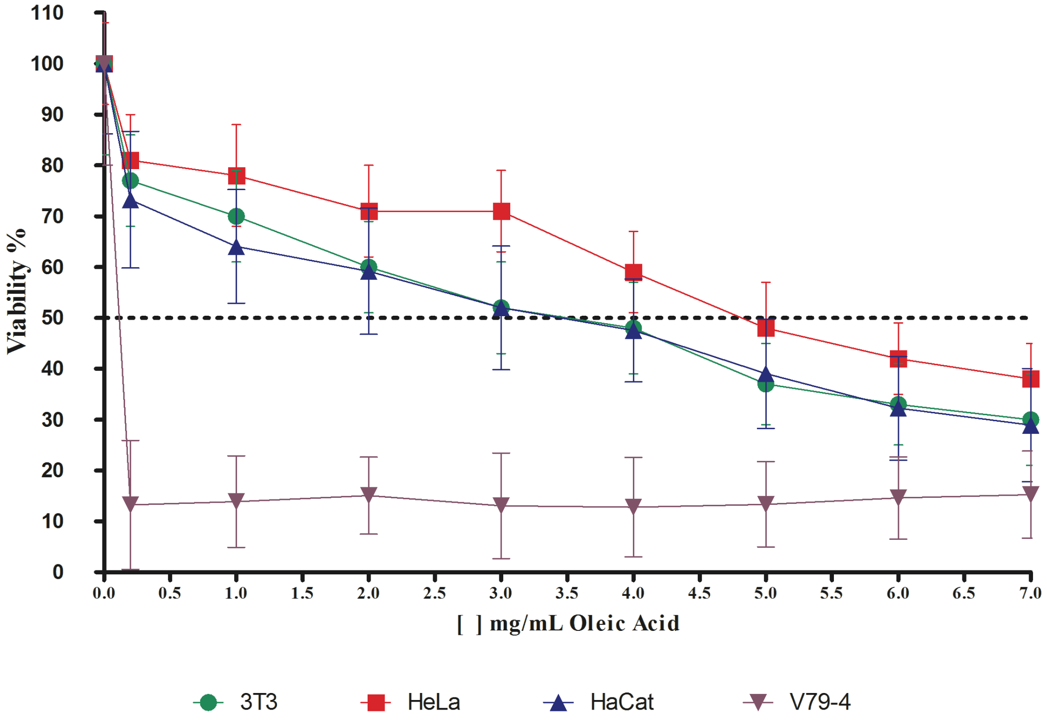

Figure 5S: Evaluation of cell viability using the MTT technique applied to the formulations containing neem oil and the different cell lines. A: 3T3; B: HeLa; C: HaCat; D: V79-4.

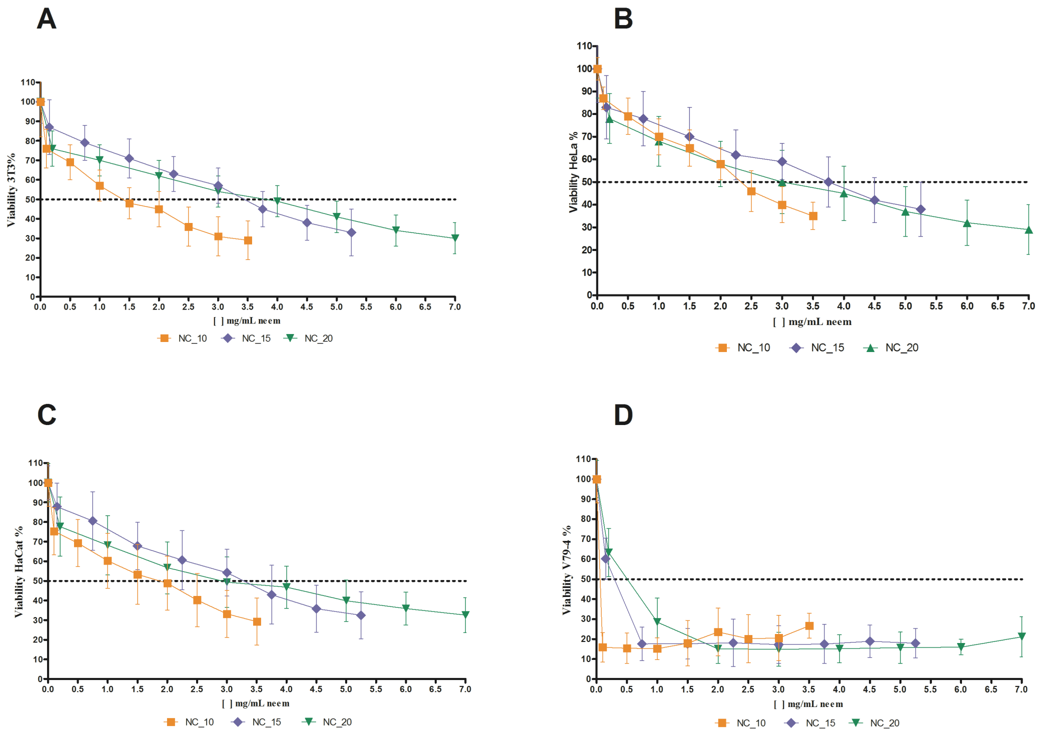

Supplement: Supplementary file 1 — Supplementary info [file 41598_2017_6092_MOESM1_ESM.pdf]
